# The Proceedings of the 16th Italian Convention of Investigators in Cystic Fibrosis: Verona, Italy. 22-24 November 2018

**DOI:** 10.1186/s40248-018-0164-1

**Published:** 2019-02-06

## Overcome the Resistance of Pseudomonas aeruginosa

### O1 Preclinical study of a host-directed therapy based on Metformin and bioactive liposomes for the control of multidrug resistant *P. aeruginosa* infection

#### Noemi Poerio^1^, Federica De Santis^1^, Alice Rossi^2^, Ana Henriquez^1^, Federica Di Sano^1^, Vincenzina Lucidi^3^, Alessandra Bragonzi^2^, Maurizio Fraziano^1^

##### ^1^Dipartimento di Biologia, Università di Roma ‘’ Tor Vergata ‘’, Roma, Italy; ^2^Unità di Infezioni e Fibrosi Cistica, Istituto Scientifico San Raffaele, Milano, Italy; ^3^Unità Operativa Complessa Fibrosi Cistica, Dipartimento di Medicina Pediatrica, Ospedale Pediatrico Bambino Gesù, Roma, Italy.

###### **Correspondence:** Maurizio Fraziano (fraziano@bio.uniroma2.it)

**Background**

We have recently showed that apoptotic body like liposomes (ABL) loaded with phosphatidylinositol 5-phosphate (PI5P) significantly enhance bactericidal response in macrophages from Cystic Fibrosis (CF) patients against *P. aeruginosa* and in bronchoalveolar lavage cells from patients with pneumonia caused by different multidrug resistance (MDR) bacterial pathogens. Moreover, Metformin (Met) has been recently reported to augment airway surface hydration, in *in vitro* models of CF, and to enhance antimicrobial innate immune response and to reduce inflammation in *in vivo* models of tuberculosis.

**Hypothesis and objectives**

The main goal of this project was the development of a novel immunotherapeutic approach based on bioactive liposome in combination with Met aimed to enhance antimicrobial innate immune response, while simultaneously improve airway surface hydration and mitigate inflammation to control multidrug resistant infections in CF.

**Methods**

Macrophages generated by peripheral monocytes derived from healthy donors, treated or not with a pharmacological inhibitor of CFTR (INH172), and from CF patients were infected with a panel of MDR *P. aeruginosa* clinical isolates and stimulated with ABL/PI5P, alone or in combinations with Met. We have evaluated intracellular bacterial clearance and uptake, and phagosome maturation by CFU and fluorimetric assays, respectively. Finally, treatments were tested in *in vivo* murine model of MDR-RP73 *P. aeruginosa* acute infection, in terms of leukocytes recruitment and bactericidal action enhancement.

**Results**

We showed that treatment with ABL/PI5P and/or Met rescues impaired phagosome acidification in CFTR-pharmacologically inhibited macrophages and promotes intracellular bacterial killing in INH172- and CF primary macrophages infected with MDR *P. aeruginosa* 2113 strain, although only ABL/PI5P stimulation increase bacterial uptake. Finally, preliminary results in *in vivo* model of MDR-RP73 *P. aeruginosa* acute infection, show that treatment with ABL/PI5P or Met induces an overall decrease in leukocytes recruitment, associated to an increase of macrophage component and to a reduction of pulmonary bacterial load, although following ABL/PI5P treatment only.

**Conclusions**

Our results show that bioactive liposome and metformin-based strategy could represent a promising host-directed therapeutic option for the control of drug resistant bacterial infections and for the reduction of the inflammation-based pathology in CF.

**Acknowledgment**

This study was supported by Italian Cystic Fibrosis Research Foundation (FFC), Research project Number FFC#14/2017

### O2 Exploiting the potential of gallium for the treatment of *Pseudomonas aeruginosa* pulmonary infection

#### Paolo Visca^1^, Francesco Peri^2^, Raffaella Sorrentino^3^

##### ^1^Dipartimento di Scienze, Lab. Microbiologia Clinica e Virologia, Università Roma Tre, Roma, Italy; ^2^Dip. Di Biotecnologie e Bioscienze, Università Milano Bicocca, Milano, Italy; ^3^Dip. Di Farmacia, Università di Napoli Federico II , Napoli, Italy

###### **Correspondence:** Paolo Visca (paolo.visca@uniroma3.it)

**Background and rationale**

Morbidity and mortality in cystic fibrosis (CF) patients is attributable to infectious sequelae caused by different pathogens[1]. Antibiotic resistance in CF calls for the development of new antimicrobials. Ga(III) inhibits bacterial growth, acting as an iron mimetic, and is already used in medicine (Ganite^®^) for treatment of non-infectious disorders[2]. Bacteria erroneously incorporates Ga(III) instead of Fe(III) within essential molecules because unable to discriminate between these two ions, resulting inhibited[3].

**Hypothesis and objectives**

The main objective of this project was a comparative assessment of the antibacterial activity of different Ga(III) formulations on major CF pathogens, and the development of safe Ga(III)-based drugs that can specifically directed in the lung of CF patients, via inhalable formulations.

**Essential methods**

We capitalized upon expertise in organic synthesis, pharmaceutical chemistry and microbiology to: i) compare the antibacterial activity of different Ga(III) formulations on major CF pathogens; ii) generate new formulations for in vivo administration and determine their pharmacological characteristics; iii) investigate acute toxicity and the organ distribution of Ga(III), upon intra-tracheal and intravenous administration in rats.

**Results**

New Ga(III) testing methods have been developed for major CF pathogens. Two compounds showed potent broad-spectrum antibacterial properties. To overcome limitations of systemic administration, a novel inhalable Ga(III)-based dry powder has been developed. The new formulation showed high Ga(III) content and stability in the long-term, good antimicrobial proprieties and an excellent biodistribution in rats after intra-tracheal aerosol administration.

**Spin-off for research & clinical purposes**

In the worrying scenario of increasing antibiotic resistance in CF-associated bacterial pathogens, Ga(III)-derived compounds are good candidates for broad-spectrum antimicrobials, and hold great promise for the progression into drugs with potential clinical applicability in the short-medium perspective.

**Acknowledgment**

This study was supported by Italian Cystic Fibrosis Research Foundation (FFC), Research project Number FFC#18/2017

**References**

1. Folkesson A, Jelsbak L, Yang L, Johansen HK, Ciofu O, Høiby N, Molin S. Adaptation of Pseudomonas aeruginosa to the cystic fibrosis airway: an evolutionary perspective. Nat Rev Microbiol. 2012; 10: 841-51.
2. Bonchi C., Imperi F., Minandri F., Visca, P., Frangipani E. Repurposing of gallium-based drugs for antibacterial therapy. Biofactors. 2014; 40; 303-312.
3. Kaneko Y., Thoendel M., Olakanmi O., Britigan B.E., Singh P.K. The transition metal gallium disrupts Pseudomonas aeruginosa iron metabolism and has antimicrobial and antibiofilm activity. J. Clin. Invest. 2007; 117; 877-888.

### O3 Phage therapy against *Pseudomonas aeruginosa* infections in a cystic fibrosis zebrafish model

#### Marco Cafora^1^, Gianluca Deflorian^2^, Francesca Forti^3^, Laura Ferrari^2^, Giorgio Binelli^4^, Federica Briani^3^, Daniela Ghisotti^3^, Anna S Pistocchi^1^

##### ^1^Dipartimento di Biotecnologie Mediche e Medicina Traslazionale – Università degli Studi di Milano – LITA – via Fratelli Cervi 93 – 20090 Segrate (MI) – Italy; ^2^Istituto FIRC di Oncologia Molecolare – IFOM – Via Adamello 16 – 20139 Milano – Italy; ^3^Dipartimento di Bioscienze - Università degli Studi di Milano - Via Celoria 26 - 20133 Milano – Italy; ^4^Dipartimento di Biotecnologie e Scienze della Vita - Università degli Studi dell’Insubria - Via J.H. Dunant 3 – Varese- Italy.

###### **Correspondence:** Anna S Pistocchi (anna.pistocchi@unimi.it)

**Background and Rationale**

We have recently isolated virulent phages capable of infecting *P. aeruginosa* and used them to treat *P. aeruginosa* infections in mouse and *Galleria mellonella* larvae. The positive outcome obtained by phage therapy encouraged us to further investigate its use in a cystic fibrosis (CF) background. Indeed, *P. aeruginosa* infections are particularly serious in CF patients and, as a consequence, CF patients are subject to frequent antibiotic treatments to control the infections. The appearance and diffusion of multidrug resistant (MDR) isolates of *P. aeruginosa* is responsible for the increasingly unsuccessful use of antibiotics and alternative therapies are urgently needed. Phages, the natural enemies of bacteria, can be a possible solution as they infect only very specific bacterial hosts, they self-control their dose multiplying only when and where the target bacterial host strains are present, and are able to kill also MDR bacteria.

**Hypothesis and Objectives**

The scientific question addressed by this work is the validation of phage therapy against *P. aeruginosa* infection in a cystic fibrosis background (CF). We chose zebrafish (*Danio rerio*) as *in vivo* model. The zebrafish model has two main advantages: it lacks an adaptive immune response for the first 4-6 weeks of life representing an ideal model for studying innate immunity and it is a good model for CF as the CFTR channel is conserved between fish and mammals.

**Essential Methods**

We deregulated the CFTR function in zebrafish, obtaining CF embryos. We infected control (WT) and CF embryos with *P. aeruginosa* and we compared lethality, bacterial burden and inflammatory cytokines after infection followed by phage administration.

**Results**

We demonstrate that phage therapy is effective against *P. aeruginosa* infections as it reduces lethality, bacterial burden and immune response in WT and in CF embryos. We also show an improvement by combining the action of phages and antibiotics against *P. aeruginosa* infection in CF zebrafish embryos. In addition, we found that phage administration, in the absence of bacterial infection, relieves the constitutive inflammatory state of CF embryos.

**Conclusions**

Our data suggest promising therapeutic approaches to reduce antibiotic doses and time of administration, avoiding the development of MDR in a CF background. To our knowledge this is the first time that phage therapy is used to cure *P. aeruginosa* infections in an *in vivo* CF model.

**Acknowledgment**

This study was supported by Italian Cystic Fibrosis Research Foundation (FFC), Research project Number FFC#22/2017

## Unexplored Areas in CF Lung Infection

### O4 Cystic fibrosis modifier genes related to Pseudomonas aeruginosa lung disease

#### Nicola Ivan Loré^1^, Claudia Caslini^1^, Gengming He^2^, Lisa Strug^2^, Migliara Alessandro^3^, Angelo Lombardo^3^, Lisa Provezza^4^, Giulio Cabrini^4^, Rizzo Giulia^1^, Barbara Sipione^1^, Emanuela Caci^5^, Louis Galietta^6^, Harriet Corvol^7^, Alessandra Bragonzi^1^

##### ^1^Infection and Cystic Fibrosis Unit, Division of Immunology, Transplantation and Infectious Diseases, IRCCS - San Raffaele Scientific Institute, Milan, Italy; ^2^Division of Biostatistics, Dalla Lana School of Public Health; University of Toronto, Toronto, Canada; ^3^San Raffaele Telethon Institute for Gene Therapy (SR-Tiget), IRCCS San Raffaele Scientific Institute, Milan, Italy; ^4^Azienda Ospedaliera Universitaria Integrata Verona, Verona Italy; ^5^ Istituto Giannina Gaslini, Genova, Italy; ^6^Telethon Institute of Genetics and Medicine, Pozzuoli, Italy; ^7^INSERM, Paris, France

###### **Correspondence:** Alessandra Bragonzi (bragonzi.alessandra@hsr.it)

**Background and rationale**

The outcomes of P. aeruginosa infections in patients with cystic fibrosis (CF) are difficult to predict and extremely variable, with the severity of the pulmonary conditions ranging from mild to life-threatening. This opens the question which other genetic loci in addition to the CFTR can contribute to the clinical variation. By using the Collaborative Cross (CC) lines as a novel and high genetically diverse mouse resource population and models of infection established by our group, we map a quantitative trait locus (QTL) on murine chromosome 3 that affect the severity of Pseudomonas aeruginosa lung infection (previous project, FFC#9/2014) .

**Hypothesis and objectives**

This project hypothesizes candidate modifier genes within the identified QTL and aims to validate them as risk factors for P. aeruginosa infection and disease severity.

**Essential methods**

Validation of candidate modifier genes was carried out: 1) in model system including gene editing with CRISPR/Cas9 of cell lines; 2) in CF patients cohorts by exploring available BIO-banks

**Results**

Within the QTL locus, 14 protein-coding genes were candidates for involvement in P. aeruginosa pneumonia. Among others, the sphingosine 1-phosphate receptor 1 (S1PR1) ranked as one of the most promising candidates. S1PR1 encodes a G-protein-coupled receptor involved in several physiological processes, including inflammation. Immunohistochemistry showed significantly decreased S1PR1 protein expression in lungs of CF patients compared with those of non-CF. Lack of S1PR1 in CF cell line (IB3) increased inflammatory response after stimulation with P. aeruginosa supernatant, indicating a possible role in the infection.

To translate our results to humans, first a genotyped cohort (Canadian CF Gene Modifier) with clinical microbiological data for P. aeruginosa infection was used for identification of candidate genes. Genetic-association analysis on the syntenic human locus on chromosome 1 identified two single-nucleotide polymorphisms annotated to the dihydropyrimidine dehydrogenase (DPYD) gene that were significantly associated with age at first P. aeruginosa infection. DPYD encoded a pyrimidine catabolic enzyme and has never been described in infection and inflammation processes. Other cohorts are under evaluation.

**Conclusions**

Our project identified possible genetic modifiers that affect the severity of P. aeruginosa lung infection.

**Acknowledgment**

This study was supported by Italian Cystic Fibrosis Research Foundation (FFC), Research project Number FFC#15/2016

## Towards New Modulators of F508del-CFTR

### O5 Development of a PI3Kγ-derived peptide as a novel F508del-CFTR potentiator

#### Alessandra Murabito^1^, Flora Pirozzi^1,2^, Kai Ren^1^, Mingchuan Li^1^, Valentina Sala^1^, Nancy L. Quinney^3^, Carlo Laudanna^4^, Martina Gentzsch^3^, Emilio Hirsch^1^ and Alessandra Ghigo^1^

##### ^1^Department of Molecular Biotechnology and Health Sciences, Molecular Biotechnology Center, University of Torino, Torino, Italy; ^2^Division of Internal Medicine, Department of Translational Medical Sciences, Federico II University, Naples, Italy; ^3^Marsico Lung Institute/Cystic Fibrosis Research Center, University of North Carolina at Chapel Hill, Chapel Hill, NC, USA; ^4^Department of Pathology and Diagnostics, Division of General Pathology, School of Medicine, Verona, Italy

###### **Correspondence:** Alessandra Ghigo (alessandra.ghigo@unito.it)

**Background and rationale**

The underlying cause of cystic fibrosis (CF) is a mutation in the CFTR gene. A number of CFTR correctors and potentiators, restoring membrane expression and cAMP-activated gating, respectively, have been developed, but their clinical efficacy is unsatisfactory [1-3]. We recently developed a peptide targeting PI3Kγ kinase-independent function [4]. (WO/2016/103176), which we found involved in the regulation of cAMP signalling. We previously demonstrated that this molecule rescues the conductance of F508del CFTR more efficiently than VX-770 potentiator.

**Hypothesis and objectives**

In the view of developing PI3Kγ peptide as a novel human medicinal product, we intended to complete the chemical optimization of our peptide lead. In particular, we sought to obtain a molecule that recapitulates the biological features of the parent PI3Kγ peptide but implying lower costs of synthesis. Finally, we further characterized the mechanism of action of the parent PI3Kγ peptide.

**Essential methods**

Peptide derivatives were screened for their ability to elevate cAMP levels in human bronchial epithelial cells (16HBE14o-). To further investigate the molecular mechanisms underlying the ability of the parent PI3Kγ peptide to rescue F508del-CFTR function, chloride current measurements and surface biotinylation assays were carried out in bronchial primary CF and CFBE41o- cells.

**Results**

The minimal active sequence (MIN seq) of PI3Kγ peptide was identified and fused to different cell penetrating peptides. We found that the efficiency of PI3Kγ MIN seq in elevating cAMP levels was comparable to that of the parent peptide when fused to Penetratin 1. On the other hand, the parent PI3Kγ peptide stimulated CFTR-dependent currents even in non-corrected primary bronchial epithelial F508del cells, suggesting that this compound may also serve as a CFTR corrector. Surface biotinylation assays in CF cells confirmed the ability of the PI3Kγ to promote trafficking of the mutant CFTR to the plasma membrane.

**Conclusions**

Overall, our data suggest that the PI3Kγ peptide exerts both corrective and potentiating effects and may be exploited as a single agent for the treatment of F508del patients. This will eventually permit to ameliorate disease management and quality of life of patients. The PI3Kγ-derived peptide received the Orphan Drug Designation by the European Medicinal Agency (EU/3/17/1859) in 2017 and we envisage to complete preclinical safety assessments in 1.5 years.

**Acknowledgment**

This study was supported by Italian Cystic Fibrosis Research Foundation (FFC), Research project Number FFC#11/2017

**References**

1. Cholon DM, Quinney NL, Fulcher ML, Esther CR Jr, Das J, Dokholyan NV, Randell SH, Boucher RC, Gentzsch M. Potentiator ivacaftor abrogates pharmacological correction of ΔF508 CFTR in cystic fibrosis. Sci Transl Med. 2014;6(246):246ra96.
2. Wainwright CE, Elborn JS, Ramsey BW. Lumacaftor-Ivacaftor in Patients with Cystic Fibrosis Homozygous for Phe508del CFTR. N Engl J Med. 2015; 373(18):1783-4.
3. Veit G, Avramescu RG, Perdomo D, Phuan PW, Bagdany M, Apaja PM, Borot F, Szollosi D, Wu YS, Finkbeiner WE, Hegedus T, Verkman AS, Lukacs GL. Some gating potentiators, including VX-770, diminish ΔF508-CFTR functional expression. Sci Transl Med. 2014; 6(246)
4. Perino A1, Ghigo A, Ferrero E, Morello F, Santulli G, Baillie GS, Damilano F, Dunlop AJ, Pawson C, Walser R, Levi R, Altruda F, Silengo L, Langeberg LK, Neubauer G, Heymans S, Lembo G, Wymann MP, Wetzker R, Houslay MD, Iaccarino G, Scott JD, Hirsch E. Integrating cardiac PIP3 and cAMP signaling through a PKA anchoring function of p110γ. Mol Cell. 2011; 42(1):84-95

### O6 Pharmacophore and pharmacokinetic filtering tools guiding for the design and synthesis of novel thiazole-containing and VX-809 hybrid derivatives as F508del correctors

#### Nara Liessi^1^, Elena Cichero^2^, Emanuela Pesce^3^, Nicoletta Pedemonte^3^, Annalisa Salis^1^, Bruno Tasso^2^, Gianluca Damonte^1,4^, Giada Righetti^2^, Paola Fossa^2^ and Enrico Millo^1,4^

##### ^1^Center of Excellence for Biomedical Research (CEBR), University of Genoa, Genoa, Italy; ^2^Department of Pharmacy, Section of Medicinal Chemistry, University of Genoa, Genoa, Italy; ^3^U.O.C. Genetica Medica, Istituto Giannina Gaslini, Genoa, Italy; ^4^Department of Experimental Medicine, Section of Biochemistry, University of Genoa, Genoa, Italy

###### **Correspondence:** Enrico Millo (enrico.millo@unige.it)

**Background**

Cystic fibrosis (CF) is an autosomal recessive genetic disorder that results from the functional deficiency in a plasma membrane anion channel known as CFTR. The disease is caused by mutations in the CFTR which encodes a cAMP-regulated chloride channel. The primary defects can be treated with drug-like small molecules, known as “CFTR modulators”. The most promising data came from molecular screening strategies, guiding towards the discovery of thiazole-containing correctors. In previous studies, we identified a class of compounds called aminoarylthiazoles (AATs) that potentially correct the CF basic defect and also showed a strong additive effect when combined with VX809.

**Hypothesis and objectives**

In our project we proceed applying a ligand-based strategy in order to enlighten the most relevant chemical groups involved in the modulator ability. Thus, we collected all the corrector chemo-types already known in the literature and used this set of molecules to perform pharmacophore analysis and QSAR studies. A limited number of selected descriptors has been chosen as filtering tools for the design of novel thiazole-based derivatives. The main objectives of our project was to identify new compounds, belonging both to the family of AATs and of VX-809 analogues, which have the ability to correct the CFTR protein defect caused by F508del.

**Essential Methods**

In an attempt to construct more active molecules, it was thought to generate chemically hybrid compounds, blending a portion of VX809 merged to the thiazole scaffold. Novel AAT analogues has been designed by filtering on the information obtained but these QSAR analyses, synthesized and tested on CFBE41o- cells expressing F508del to further explore the chemical space around the thiazole ring.

**Results**

We evaluated different AAT-VX-809 hybrid derivatives with corrector properties. The new molecules were tested in functional and biochemical assays showing a promising corrector activity. Starting from the most active compounds, we have designed a second series of hybrids that could improve the good results obtained.

**Conclusions**

Herein we reported a ligand-based approach including quantitative-structure activity relationship (QSAR) that efficiently led to the rational design and optimization of VX- 809 and thiazole-containing hybrid compounds. Such molecules may represent lead compounds for the development of drugs that correct the basic defect in CF patients.

**Acknowledgment**

This study was supported by Italian Cystic Fibrosis Research Foundation (FFC), Research project Number FFC#6/2017

### O7 Myriocin potential as a phenotype-modifying therapeutical in Cystic Fibrosis

#### Alessandra Mingione^1,3^, Emeranziana Ottaviano^2^, Fabiola Bonezzi ^3^, Michele dei Cas ^3,4^, Marco Piccoli ^5^, Matteo Barcella ^2^, Anna Caretti ^3^, Rita Paroni ^4^, Riccardo Ghidoni ^3^, Natalia Cirilli^6^, Elisa Borghi ^2^, Paola Signorelli ^3^

##### ^1^Italian Research Cystic Fibrosis Foundation, Verona, Italy; ^2^Microbiology Laboratory, Health Sciences Department, University of Milan, Milan Italy; ^3^Biochemistry and Molecular Biology Laboratory, Health Sciences Department, University of Milan, Milan, Italy; ^4^Clinical Biochemistry and Mass Spectrometry Laboratory, Biochemistry and Molecular Biology Laboratory, Health Sciences Department, University of Milan, Milan, Italy; ^5^Stem Cells for Tissue Engineering Laboratory, IRCCS Policlinico San Donato, Italy; ^6^Cystic Fibrosis Regional Reference Center, United Hospitals, Salesi Childrens’ Hospital Mother, Child Department, Ancona, Italy

###### **Correspondence:** Paola Signorelli (paola.signorelli@unimi.it)

**Background and rationale**

Deletion of phenylalanine 508 (ΔF508) in CFTR gene, occurring in 70% of Cystic Fibrosis (CF) patients, induces a proteinopathy that is characterized by aggregates of mutated proteins, hyper-inflammation, impaired trafficking and altered metabolism at cellular level. Aside to lung chronic inflammation and infections, severe comorbidities develop in pancreas, kidney, include infertility, osteopenia, diabetes and dyslipidemia with increased plasma triglyceride and tissues cholesterols, pancreas fibrolipomatosis, hepatic lipogenesis and steatosis [1]. The lipotoxin ceramide contributes to lung hyper-inflammation [2]. We previously demonstrated that the ceramide synthesis inhibitor Myriocin,reduces inflammation and ameliorates defense response against bacterial and fungal infection in CF *in vitro* and *in vivo* models [3,4].

**Hypothesis and objective**s

We here aim at demonstrating the mode of action of Myriocin as an antiflammatory and antimicrobial therapeutic agent.

**Essential Methods**

Myriocin treatment will be evaluated in ΔF508-CFTR broncho epithelial cell line and in peripheral blood monocytes derived from CF patients, either homozygous or heterozygous for ΔF508.

**Results**

First, we show that Myriocin is an effective inducer of autophagy, which is defective in CF. Second, we demonstrate that Myriocin activates key transcriptional factors, TFEB, FOXO1a and PPARgamma, involved in autophagy induction, mitochondrial activity, energy production, lipid mobilization and consume. Next, we prove that Myriocin significantly increases the transcription of downstream genes, regulating fatty acids entry in mitochondria (CTP1a and 1b; FATP) and their oxidation (ACAD L). We show that Myriocin significantly reduces pathological accumulation of lipid un-organized deposits. We observe that inhibition of sphingolipid synthesis causes a reduced content of non sphingoid-lipids such as cholesterols. By RNA sequencing, we then prove that Myriocin changes the transcriptional profile of treated cells, enhancing the transcription of genes involved in lipid transport and consume and energy metabolism, that is partially downregulated in CF. Finally Myriocin treatment of peripheral blood monocytes from CF patients, infected with *A. fumigatus*, significantly increases their pathogen killing ability.

**Conclusions**

Myriocin has a therapeutic action against infection and it is a promising agent in the cure of CF diabetes and dyslipidemia comorbidities.

**Acknowledgment**

This study was supported by Italian Cystic Fibrosis Research Foundation (FFC), Research project Number FFC#11/2016

**References**

1. Figueroa V, Milla C, Parks EJ, Schwarzenberg SJ, Moran A. Abnormal lipid concentrations in cystic fibrosis. Am J Clin Nutr. 2002;75(6):1005-11.
2. Teichgräber V, Ulrich M, Endlich N, Riethmüller J, Wilker B, De Oliveira-Munding CC, van Heeckeren AM, Barr ML, von Kürthy G, Schmid KW, Weller M, Tümmler B, Lang F, Grassme H, Döring G, Gulbins E. Ceramide accumulation mediates inflammation, cell death and infection susceptibility in cystic fibrosis. Nat Med. 2008;14(4):382-91.
3. Caretti A, Torelli R, Perdoni F, Falleni M, Tosi D, Zulueta A, Casas J, Sanguinetti M, Ghidoni R, Borghi E, Signorelli P. Inhibition of ceramide de novo synthesis by myriocin produces the double effect of reducing pathological inflammation and exerting antifungal activity against A. fumigatus airways infection. Biochim Biophys Acta. 2016;1860(6):1089-97.
4. Caretti A, Bragonzi A, Facchini M, De Fino I, Riva C, Gasco P, Musicanti C, Casas J, Fabriàs G, Ghidoni R, Signorelli P. Anti-inflammatory action of lipid nanocarrier-delivered myriocin: therapeutic potential in cystic fibrosis. Biochim Biophys Acta. 2014;1840(1):586-94.

## Clinical Perspectives

### O8 Outcomes of spontaneous application of carrier screening for cystic fibrosis: follow-up of its effects on birth prevalence, neonatal screening and reproductive behavior of carrier couples

#### Carlo Castellani (carlocastellani@gaslini.org)

##### Centro FC, Istituto G. Gaslini, Genova (Italy)

**Background**

In the latest decades the offer of CF carrier tests to individuals and couples with non increased *a priori* risk of having affected children (*Carrier Screening*) has been widely practiced in a sub-area of north-eastern Italy (Eastern Veneto), although not in a structured and formal setting. A reverse correlation between number of carrier tests and CF incidence was previously shown.

**Hypothesis and objectives**

This observational study aimed at 1) understanding if a drop in the use of carrier screening could be associated with higher CF incidence, 2) monitoring trends of CF birth prevalence in the eastern part of the Veneto region over an unprecedented long period; 3) evaluating reproductive behavior of carrier couples identified by carrier screening.

**Methods**

- Collection of data on CF tests performed, quality control and monitoring of results obtained (source: genetic labs).
- Collection of data on CF birth prevalence (source: neonatal screening of the Verona CF Centre.
- Investigation on the causes of the decrease in the number of tests performed in the Study Area.
- Survey on CF Genetic Counseling sessions performed (2014-2016) in an experimental subarea, with the aim of evaluating the ways of access to counseling of carriers and carrier couples.

**Results**

- Collection of data on Carrier Screening completed (1993-2017).
- A reduction in the number of tests performed in the area is confirmed, with a concomitant increase in CF incidence.
- Identification of the main causes of the test reduction, namely:1)effects of the economic crisis; 2) reorganization of the carrier test offer following the entry into force of the pertaining recent regional legislation; 3) consequent transformation of the private supply market.
- Re-evaluation of carrier frequency in the area under study: 1/29
- Only a fraction of carriers detected by screening use genetic counseling facilities.

**Conclusions**

- The association between more newborns with CF and less carrier tests support previous data on the correlation between carrier screening and CF incidence.
- The results of the Survey on Genetic Counseling indicate the importance of implementing structured carrier screening programs that provide adequate access to pre-test information and consulting after analysis.

**Acknowledgment**

This study was supported by Italian Cystic Fibrosis Research Foundation (FFC), Research project Number FFC #26/2015

### O9 Italian multicenter study of glucose tolerance defects in cystic fibrosis

#### Alberto Battezzati ^1^, Carla Colombo^2^, Vincenzina Lucidi ^3^, Giuseppe Magazzù ^4^, Andrea Mari^5^

##### ^1^DeFENS, Università di Milano**,** Milano, Italy; ^**2**^Centro FC, Policlinico Mangiagalli, Milano**,** Italy; ^**3**^Ospedale Bambino Gesù, Roma**,** Italy; ^**4**^Università di Messina, Messina, Italy; ^**5**^IN, CNR, Padova, Italy

###### **Correspondence:** Alberto Battezzati (alberto.battezzati@unimi.it)

**Background and rationale**

Cystic Fibrosis Related Diabetes (CFRD) is a frequent complication associated to pulmonary and nutritional decay even years prior to diagnosis. Prominent defect is insufficient insulin secretion worsening with age. From 2003 we followed prospectively more than 300 patients with 1,000 OGTTs (Oral Glucose Tolerance Test) modified to quantify insulin secretory and sensitivity defects.

**Hypothesis and objectives**

Insulin secretory defects arise early in life, may predispose to CFRD, nutritional and respiratory deterioration. Focus is on the deleterious effects of reduced insulin secretion prior to the development of hyperglycemia. Primary aim was to create for the Italian CF population, reference values of glucose, insulin and c-peptide concentrations in response to OGTT, and of model-derived parameters of insulin secretion and insulin sensitivity. Secondarily, to clarify the relationship of secretory defects with pancreatic insufficiency, liver disease and new CF therapies

**Essential methods**

a) creation of a centralized data platform to upload all clinical and laboratory data, suitable to follow up b) 450 OGTTs with simultaneous measure of insulin secretory and sensitivity parameters c) reference values calculation at a national level d) analysis of the association of the secretory parameters with clinical endpoints

**Results**

For the OGTTs performed, funding covered the collection of glucose concentrations and clinical data, but co-funding from recruiting centers allowed the completion of 406 studies with insulin secretory data and the remaining studies being concluded with insulin secretory data shortly. Insulin and c-peptide assays provided comparable measures among centers. Modelling is underway as well as statistical analysis for reference values and relationship with clinically relevant biomarkers. Preliminary analysis from the Milan group (inclusive of previously collected data) showed that Glucose Sensitivity and Insulin concentration at 30’ are the main correlates of lung function (1) and long term predictors of CFRD and adult height

**Conclusions**

Sex and age adjusted nomograms of OGTT parameters will be powerful tools to describe the glucose tolerance defects natural history, to predict future CFRD, nutritional and respiratory decay and to set a rational basis for treatment. A collaboration has been set with a US multicenter group, that will replicate our protocol and analysis in an equal number of patients, under the agreement to produce common international reference values.

**Acknowledgment**

This study was supported by Italian Cystic Fibrosis Research Foundation (FFC), Research project Number FFC#20/2016

### O10 Environmental and human reservoirs of *Pseudomonas aeruginosa* and other bacterial species colonizing the lower airways of cystic fibrosis patients

#### Caterina Signoretto (caterina.signoretto@univr.it)

##### Department of Diagnostic and Public Health, Microbiology Section, University of Verona, Verona, Italy

**Background and rationale**

There are evidence that the upper airway (nose, paranasal sinuses) and oral cavity may be reservoir of *Pseudomonas aeruginosa* (Pa) and other bacterial that cause infections in CF lung [1,2]. These adaptation sites probably allow bacteria to persist and then colonize the lower airways. Monitoring and eradication of potentially pathogenic nasal and oral microflora in FC patients is therefore mandatory to prevent/delay a chronic lung infection [3,4].

**Objectives**

1. To set up and validate a protocol for the recovery of bacterial nasal microflora by nasal lavage (NAL) both in adults and children CF.
2. To evaluate the incidence of potentially pathogenic bacterial species in the upper airway/oral cavity and compare them with those isolated from the sputum.
3. Comparison, by molecular profiles, of bacteria isolated in the upper and lower respiratory tract to confirm their recirculation between the two anatomical districts
4. Evaluation of the possibility that toothbrushes may act as bacterial reservoirs favouring the colonization of the oral cavity.

**Case report** A total of 60 patients (adult and pediatric) were enrolled; they were divided into two groups: free of colonization of Pa and with chronic infection of Pa. All patients are followed for 12/14 months with visits and sampling of NAL and sputum for a total of 2/3 visits per patient. The Pa free patients, were also involved in monitoring the bacterial flora in the oral cavity and toothbrushes with saliva sampling.

**Conclusion**

1. Patients Pa chronic infection: colonization in the lung prevent colonization by other bacterial species, indeed no other microorganism was isolated.
2. Patients no chronic Pa infection: colonization by differents emerging pathogens for which there is still no clear pathogenic role.
3. Pediatric patients: *S.aureus* is the predominant bacterial species and it is often isolated together with other bacterial
4. A high percentage of patients are colonized in the nasal and pulmonary sites by the same genetically related bacterial species, indicating that it is the same clone and confirms their passage from the high to the lower airways.
5. About 40% of toothbrushes were colonized by bacteria and carry them in the oral cavity and thus potentially in the lower respiratory tract.

**Clinical purposes**

- Proposal of guidelines for collection and monitoring of the bacterial flora of the nasal/ paranasal reservoir of selected patients FC, by application of NAL sampling
- Identify therapeutic protocols for eradication of upper respiratory tract bacteria in order to prevent their passage in the lower respiratory tract and avoid/delay the onset of chronic lung infection
- Introduction of treatment and conservation protocols of toothbrushes in order to avoid contact with environmental bacteria.

**Acknowledgment**

This study was supported by Italian Cystic Fibrosis Research Foundation (FFC), Research project Number FFC#22/2016

**References**

1. Sobin L, Kawai K, Irace AL, Gergin O, Cunningham M, Sawicki GS, Adil EA. Microbiology of the Upper and Lower Airways in Pediatric Cystic Fibrosis Patients. Otolaryngol Head Neck Surg. 2017;157(2):302-308
2. Berkhout MC, Rijntjes E, El Bouazzaoui LH, Fokkens WJ, Brimicombe RW, Heijerman HG**.** Importance of bacteriology in upper airways of patients with Cystic Fibrosis. J Cyst Fibros 2013; 12(5): 525-9.
3. Wilson P, Lambert C, Carr SB, Pao C. Paranasal sinus pathogens in children with cystic fibrosis: do they relate to lower respiratory tract pathogens and is eradication successful? J Cyst Fibros. 2014; 13(4):449-54
4. Franzelle, MR, and Munro CL. Toothbrush contamination: a review of the literature. Nursing Research and Practice Volume 2012; Article ID 420630.

### O11 *Pseudomonas aeruginosa* eradication in patients with cystic fibrosis: a randomised multicentre study comparing classic treatment protocols with classic treatment together with antibiotic treatment of upper airways

#### Giovanni Taccetti^1^, Daniela Dolce^1^, Silvia Campana^1^, Novella Ravenni ^1^, Michela Francalanci ^1^, Gianfranco Mergni^1^, Tommaso Orioli^1^, Lucia Zavataro^1^, Cesare Braggion^1^, GiuseppeTuccio^2^, Carla Colombo^3^, Lisa Cariani ^4^, Daniela Girelli^4^, Mirella Collura^5^, Maria Antonietta Orlando^5^, Giovanna Pisi^6^, Maria Luisa Villani^6^, Francesco Longo^6^

##### ^1^Centro Fibrosi Cistica AOU Meyer, Firenze, Italy; ^2^Centro Regionale di Riferimento per la Fibrosi Cistica Regione Calabria, Catanzaro, Italy; ^3^Centro Fibrosi Cistica, Università di Milano, Fondazione IRCCS, Ca' Granda Ospedale Maggiore Policlinico, Milano, Italy; ^4^Laboratorio di Microbiologia, Fondazione IRCCS, Ca' Granda - Ospedale Maggiore Policlinico, Milano, Italy; ^5^Centro Fibrosi Cistica Palermo, Palermo, Italy; ^6^Centro Fibrosi Cistica Parma, Palermo, Italy

###### **Correspondence:** Giovanni Taccetti (g.taccetti@meyer.it)

**Background**

Chronic pulmonary infection due to *P. aeruginosa* is a negative prognostic factor for cystic fibrosis (CF) patients. The eradication of *P. aeruginosa* in CF patients has been one of the major areas of treatment success in the last 10 years. Early antibiotic treatment can eliminate the bacteria in 70% of cases and delay the development of chronic infection. Currently, there is no gold standard treatment protocol.

**Hypothesis and objectives**

It has been demonstrated that patients can be reinfected by *P. aeruginosa*. Recent data indicate that the paranasal sinuses are an initial site of the infection and serve as a reservoir for subsequent reinfection. It has also been hypothesized that P. *aeruginosa* undergoes genetic adaptation in the respiratory tract of CF patients.

The main objective of this study is to compare the efficacy of two types of treatment: the classic eradication protocol used until now, versus the classic protocol together with nasal lavage with colistin. The role of the paranasal sinuses in the development of P. *aeruginosa* infection will be studied microbiologically.

**Methods**

CF patients were randomised to receive either the classic or the experimental treatment (duration is 4 weeks in both groups). Eradication is defined as 3 negative, successive *P. aeruginosa* cultures within 6 months. The P. *aeruginosa* strains isolated both from the sinuses and the lower respiratory tract of the patients will be investigated microbiologically to evaluate genetic mutations.

**Results**

51 patients were randomised in 5 Centres, 25 males and 26 females (average age 13.2 ± 9.8 years). 20% of patients had positive *P. aeruginosa* samples in the upper airway at the time of enrolment. The mean FEV1 values ​​at recruitment were 79.5 ± 19.7 (% of predicted). 25 patients were randomized to classic treatment and 26 to classic treatment associated with nasal irrigations with colistin. 47 (92%) of 51 patients completed 6 months of follow-up with 3 culture tests (follow-up is still ongoing in 4 patients). At present, the group of patients undergoing experimental treatment has a reduced risk of permanence of *P. aeruginosa* in the lower airways compared to the group of patients in the comparative arm (OR = 0.47 95% CI = 0.10-2.20).

In patients with recolonization of the lower airways within 6 months the responsible strain showed an identical genotype.

**Conclusions**

At the present time follow-up in 4 patients must be completed. Although the power of the study is limited, the use of nasal lavages with colistin, in association with classical eradication strategies, was well accepted and could reduce the risk of permanence of *P. aeruginosa* in the lower airway in CF patients. Molecular studies on *P. aeruginosa* strains are still ongoing.

**Acknowledgment**

This study was supported by Italian Cystic Fibrosis Research Foundation (FFC), Research project Number FFC#30/2015

## New Targets and Rescue Mechanisms of F508-del CFTR

### O12 Understanding and targeting the regulatory pathways that control F508del-CFTR proteostasis

#### Raman Parashuraman, Advait Subramanian, Ramanath Hegde and Alberto Luini

##### IBP, CNR, Naples (Italy)

###### **Correspondence:** Alberto Luini (a.luini@ibp.cnr.it)

**Background and rationale**

Cystic fibrosis (CF) is caused by mutations in CF transmembrane conductance regulator (CFTR). The most frequent mutation (F508del-CFTR) results in the misfolding of the mutant protein that leads to its intracellular retention and degradation. Several corrector compounds have been identified that restore the proteostasis of the mutant protein. We recently identified the molecular changes associated with these corrector drugs that contributed to the rescue of the mutant protein from degradation. These changes included modulation of the activity of several signaling pathways and also ubiquitin ligases. The MLK3 pathway. Among the signaling pathways, one centered on Mixed Lineage Kinase 3 (MLK3) potently controlled the proteostasis of CFTR. We are analyzing and targeting these pathways with suitable drugs, when possible.

**Hypothesis and objectives**

We propose that MLK3 pathway acts on CFTR proteostasis by modulating the levels of machinery controlling proteostasis and small molecules targeting this pathway signalling can be potent correctors of F508del-CFTR proteostasis. So here we identify the molecular mechanism of action of this pathway and also identify repositionable drugs targeting this pathway as correctors to rescue F508del-CFTR.

**Essential methods**

1) We have used transcriptional profiling (microarrays or RNA-seq) under conditions when the MLK3 pathway was downregulated to understand the molecular changes that can be correlated with the rescue of F508del-CFTR.

2) We have identified appropriate small molecules or combinations of them targeting MLK3 pathway and rescue F508del-CFTR in model systems.

**Results**

*MLK3 pathway.* By transcriptional profiling we identified Insulin induced gene – 1 (INSIG-1) to be an essential downstream executor of the proteostasis control by MLK3 pathway. Specifically, we found that INSIG-1 binds to CFTR and targets it to endoplasmic reticulum associated degradation possibly via the ubiquitin ligase gp78. INSIG-1 is known to regulated the proteostasis of its client proteins by binding to Sterol sensing domain (SSD) present in these proteins. We found that CFTR has domain with similarities to SSD on its second transmembrane domain. By mutating a key proline residue in this domain, we find that F508del-CFTR lost its sensitivity to INSIG-1 expression. The intersection of the cholesterol homeostasis regulated by INSIG-1 and the CFTR proteostasis is currently being investigated. We also screened several clinically approved (or safety approved after crossing Phase I trials) MLK3 pathway inhibitors as potential correctors of F508del-CFTR proteostasis and found 3 active compound that restored proteostasis of F508del-CFTR as measured by biochemical maturation assays. Among these 3 drugs, one of them restored also the chloride conductance in the F508del-CFTR expressing cells to levels comparable to that of VX-809.

*The ubiquitin ligases.* In addition, we also evaluated the mechanism of action of RNF215, an uncharacterized ubiquitin ligase identified in our earlier analysis, whose depletion exert a potent corrector activity. We found that RNF215 directly binds to F508del-CFTR to target it to degradation. RNF215 downregulation also potently restored the chloride conductance in the F508del-CFTR expressing cells.

**Expected final results and their significance**

These analyses provide insights into CF pathology; provide specific targets to control F508del-CFTR proteostasis and also efficient small molecules regulators that can rescue F508del-CFTR. The approach precisely encapsulates the mission of CF foundation to develop innovative pharmacological approaches to correct or compensate the deficiency of functional CFTR.

**Acknowledgment**

This study was supported by Italian Cystic Fibrosis Research Foundation (FFC), Research project Number FFC#6/2016

### O13 Identification of the binding sites of CFTR correctors

#### Debora Baroni, Giulia Amico, Oscar Moran

##### IBF – CNR, Genova (Italy)

###### **Correspondence:** Oscar Moran (oscar.moran@cnr.it)

**Background and rationale**

Cystic fibrosis (CF) is caused by dysfunction of the CF Transmembrane conductance Regulator (CFTR), a cAMP-regulated anion channel that resides in the apical membrane of epithelial cells. CFTR dysfunction can occur by defects in protein synthesis, folding, intracellular trafficking, channel gating, conductance, or plasma membrane stability. In each case, loss of CFTR results in abnormalities of water, chloride, and bicarbonate transport that lead to dysfunction of target tissues. In the Caucasian population, the deletion of phenylalanine at position 508 of the protein (F508del) is the most frequent mutation. It causes errors in folding, trafficking and docking of the protein, determining the lack of expression of functional CFTR at the plasma membrane. In recent years some correctors, able to rescue the defects of the F508del-CFTR, partially increasing its functional expression in the plasma membrane, have been identified.

**Hypothesis and objective**

Despite the encouraging drug discovery results, to date no corrector mechanism of action and no corrector binding site have been defined. For this reason, in this project a big effort was addressed to the identification of correctors’ binding sites.

**Essential methods**

To achieve results, we prepared constructs codifying for WT and mutant F508del N-half of CFTR and expressed them alone or together with the C-half in mammalian cells. Cells preparations were incubated with different correctors (VX809, VX661, corr-4a and VX325) to evaluate the effect of each drug on the expression and stability of the two CFTR halves.

**Results**

Our results show that expression and stability of WT N-half of the CFTR protein resulted enhanced by correctors VX809, VX661 and VX325, while only VX809 and VX661 demonstrated able to exert this effect on F508del N-half. Co-expression experiments indicated that the C-half of the CFTR is the main target of corr-4a. Indeed, it significantly enhanced the expression as well as the stability of this polypeptide.

**Conclusions**

Retrieved results confirm that we are able to quantitatively evaluate the effects of a corrector on each CFTR domain. Complimentary approaches such as competition and cross-linking experiments will definitively permit us to identify the binding sites of available correctors and provide useful information for the design of better corrector candidates.

**Acknowledgment**

This study was supported by Italian Cystic Fibrosis Research Foundation (FFC), Research project Number FFC#8/2016

### O14 Alternative strategies for F508del-CFTR repair: novel targets for drug discovery approach in cystic fibrosis

#### Giorgio Cozza^1^, Antonella Tosco^2^, Eleonora Ferrari^3^, Valeria Rachela Villella^2^, Speranza Esposito^3^, Romina Monzani^3^, Mara Cetty Spinella^3^, Andrea Venerando^4^, Fabiola De Gregorio^2^, Monica Rossetto^1^, Valentina Bosello Travain^1^, Fulvio Ursini^1^, Valeria Raia^2^, Luigi Maiuri^3^

##### ^1^Department of Molecular Medicine, University of Padua, Padua, Italy; ^2^Department of Translational medical sciences, University of Naples Federico II, Regional Cystic Fibrosis Care Unit, Naples, Italy; ^3^European Institute for Research in Cystic Fibrosis (IERFC), San Raffaele Institute, Milan, Italy; ^4^Department of Comparative Biomedicine and Food Science, University of Padua, Viale dell’Università 16, 35020 Legnaro (PD), Padua, Italy

###### **Correspondence:** Giorgio Cozza (giorgio.cozza@unipd.it)

**Background and Rationale**

Cystic Fibrosis (CF) is a life-shortening genetic disorder, caused by mutations of Cystic Fibrosis Transmembrane-conductance Regulator (CFTR) [1]. The most common F508delCFTR mutant is unable to traffic to and reside at the plasma membrane (PM) [2]. Currently, CFTR-repairing therapies available for F508delCFTR are not very effective. For this reason our novel approach aims at targeting the derailed CF intracellular environment, and not directly the mutant CFTR, through a combination of drugs (e.g. cysteamine and epigallocatechin-gallate, EGCG) able to inhibit TG2 activity and specific protein kinases [3].

**Hypothesis and Objectives**

We aim to 1) refine new targets as novel therapeutic strategy in CF by exploiting a network of in silico and experimental approaches; 2) validate the efficacy of novel drug candidates in pre-clinical CF models.

**Essential Methods**

We used: A) in silico approaches to identify novel chemical entities able to interact with our new protein targets [4]; B) in vitro and in cell methodologies to validate the best candidates from A; C) in vivo validation into appropriate mouse models.

**Results**

A) Refining cysteamine structure: the chemical optimization of cysteamine led to a series of novel compounds (CT-family), active in restoring F508delCFTR function, up to more than 50% respect to control. In particular, CT11 is the best optimized molecule, being able to restore CFTR function equally to cysteamine (>70%), at a concentration more than 500 fold lower (0.1 μM vs 250 μM). These results were obtained via SPQ assay, in both CFBE41o- cells and ex vivo in cells collected by nasal brushing from CF patients. Accordingly with CT11 results on CFTR function, immunoblot detection of CFTR confirm that a sizeable fraction of the channel resides at the PM after treatment with 0.1 μM CT11. Moreover CT11 (0.1μM) is able to restore Beclin 1-dependent autophagy and to reduce P62, decreasing at the same time the inflammation biomarkers (phospho)p42/44 MAPK. Finally, CT11 is able to rescue the CFTR protein and function as well as restablish the autophagy pathway in F508del-Cftr homozygous (CftrF508del\F508del) mice, a result obtained at lower concentration compare to cysteamine and effective after 20d of wash out.

B) Identification of new approved lead compounds active in restoring F508delCFTR function: by applying our discovery strategies against our target models, we have focused our attention on our database of approved chemical entities, with the aim to preliminary identify molecule potentially transferable to the clinical use. A small number of approved entities were identified and prioritized for experimental validation via SPQ assay in CFBE41o- cells. Intriguingly, three compounds showed encouraging results, compared to cysteamine. Indeed, AMX, CT47 and PTE were able to restore CFTR function similarly to cysteamine, but at a lower concentration (25, 50 and 10μM, respectively). These results obtained unequivocally demonstrate that our research strategy is indeed very solid and that these lead molecules (after further studies ex vivo and in vivo) could represent a very promising resource for future developments and applications in Cystic Fibrosis.

**Conclusion**

The novel compounds identified represent a new frontier for the treatment of Cystic Fibrosis, by a) clarifying the roles of several other protein targets in CF, despite from the F508delCFTR itself; b) paving the way for novel phase clinical studies with a combination of molecules (or a single drug candidate) able to improve the life of CF patients.

**Acknowledgment**

This study was supported by Italian Cystic Fibrosis Research Foundation (FFC), Research project Number FFC#10/2017

**References**

1. De Boeck K, Zolin A, Cuppens H, Olesen HV, Viviani L. The relative frequency of CFTR mutation classes in European patients with cystic fibrosis J Cyst Fibros. 2014;13(4):403-9.
2. Jones AM, Barry PJ. Lumacaftor/ivacaftor for patients homozygous for Phe508del-CFTR: should we curb our enthusiasm? Thorax. 2015;70(7):615-6.
3. De Stefano D, Villella VR, Esposito S, Tosco A, Sepe A, De Gregorio F, Salvadori L, Grassia R, Leone CA, De Rosa G, Maiuri MC, Pettoello-Mantovani M, Guido S, Bossi A, Zolin A, Venerando A, Pinna LA, Mehta A, Bona G, Kroemer G, Maiuri L, Raia V. Restoration of CFTR function in patients with cystic fibrosis carrying the F508del-CFTR mutation. Autophagy. 2014;10(11):2053-74.
4. Cozza G, Bonvini P, Zorzi E, Poletto G, Pagano MA, Sarno S, Donella-Deana A, Zagotto G, Rosolen A, Pinna LA, Meggio F, Moro S. Identification of ellagic acid as potent inhibitor of protein kinase CK2: a successful example of a virtual screening application. J Med Chem. 2006;49(8):2363-6.

### O15 Modulation of protein kinases in the regulation of chaperone machinery leading F508-del-CFTR fate

#### Claudio D’Amore^1^, Christian Borgo^1^, Luca Cesaro^1^, Valentina Salizzato^1^, Valentina Bosello-Travain^2^, Andrea Venerando^3^, Mauro Salvi^1^

##### ^1^Department of Biomedical Sciences, University of Padova, Via U. Bassi 58/B, Padova, Italy; ^2^Department of Molecular Medicine, University of Padova, Via Gabelli 63, Padova, Italy; ^3^Department of Comparative Biomedicine and Food Science, University of Padova, Viale dell'Universita' 16, Legnaro, Padova, Italy

###### **Correspondence:** Mauro Salvi (mauro.salvi@unipd.it)

**Background and Rationale**

Deletion of phenylalanine 508 in CFTR (F508del) is the most common mutation in Cystic Fibrosis (CF) patients (70-90%). F508delCFTR maintains channel activity, but the mutation causes the majority of CFTR protein to be retained in the endoplasmic reticulum and prematurely degraded by the ubiquitin-proteasome system before it reaches the plasma membrane. The combination of lumacaftor-ivacaftor (corrector plus potentiator, Orkambi^TM^) represents the first therapy for CF patients homozygous for F508del-CFTR mutation, and in general a combination of therapy seems to show improved clinical benefits over available monotherapies [1].

Independent reports have shown that the heterotetrameric (two catalytic α or α’ and two β regulatory subunits) ubiquitous and constitutively active Ser/Thr protein kinase CK2 is linked to CFTR channel function. Recently CK2 inhibition have been proposed in combinatory therapy with the proteostasis regulator cysteamine [2]. However, the knowledge of the molecular mechanism(s) linking CK2 and F508delCFTR are lacking.

**Hypothesis and objectives**

Recently it has been demonstrated that CK2 targeting could be a viable strategy to reduce the expression of HSP27 [3], a member of the small heat shock proteins involved in F508delCFTR degradation [4]. To investigate the possible role of CK2 as a potential therapeutic target for treat F508del patients we analyzed firstly if CK2 signaling in these patients is deregulated, as suggested by previous reports, and secondly if CK2-targeting via HSP27 downregulation could be a valuable strategy to increase F508delCFTR plasma membrane trafficking and/or its stability.

**Essential Methods**

Both CFBE41o^−^ cell variant, stably expressing either wild-type CFTR (CFBE-WT) or the mutant F508delCFTR (CFBE-ΔF) (provided by J. P. Clancy), and primary human cells from healthy and F508delCFTR patients, purchased from the Lab. Genetica Molecolare, Istituto Giannina Gaslini (Genova), were used in this study. CFBE-ΔF cells knockout for CK2 catalytic subunits or for HSP27 have been generated by Crispr/Cas9 gene editing tool. F508delCFTR functional recovery was assayed by western blotting and by SPQ analysis.

**Results**

Our results show that, in contrast with what is observed in immortalized cell lines, CK2 expression and activity is not specifically impaired in F508delCFTR patients. In addition, we have analyzed the contribution of CK2 targeting in combination with different type of correctors or proteostasis regulator in our CK2 knockout cell models obtaining only modest results despite an overall reduction of HSP27 expression. On the contrary, HSP27 knockout CFBE cells show an improved response to correctors treatments leading to a strong functional recovery of F508delCFTR.

**Conclusions**

Whilst on the one hand these pre-clinical studies minimize the effectiveness of CK2 as a potential target in treating F508delCFTR patients, on the other they provide experimental evidences that HSP27 targeting could be a valuable strategy in a combinatory therapy with Vertex compounds.

**Acknowledgment**

This study was supported by Italian Cystic Fibrosis Research Foundation (FFC), Research project Numbers FFC#10/2016 and FFC#12/2017

**References**

1. Veit G, Avramescu RG, Chiang AN, Houck SA, Cai Z, Peters KW, Hong J6, Pollard HB, Guggino WB, Balch WE, Skach WR, Cutting GR, Frizzell RA, Sheppard DN, Cyr DM, Sorscher EJ, Brodsky JL, Lukacs GL. From CFTR biology toward combinatorial pharmacotherapy: expanded classification of cystic fibrosis mutations. Mol Biol Cell. 2016;27(3):424-33.
2. De Stefano D, Villella VR, Esposito S, Tosco A, Sepe A, De Gregorio F, Salvadori L, Grassia R, Leone CA, De Rosa G, Maiuri MC, Pettoello-Mantovani M, Guido S, Bossi A, Zolin A, Venerando A, Pinna LA, Mehta A, Bona G, Kroemer G, Maiuri L, Raia V. Restoration of CFTR function in patients with cystic fibrosis carrying the F508del-CFTR mutation. Autophagy. 2014; 10(11):2053-74.
3. Borgo C, Vilardell J, Bosello-Travain V, Pinna LA, Venerando A, Salvi M. Dependence of HSP27 cellular level on protein kinase CK2 discloses novel therapeutic strategies. Biochim Biophys Acta Gen Subj. 2018;1862(12):2902-2910.
4. Ahner A, Gong X, Schmidt BZ, Peters KW, Rabeh WM, Thibodeau PH, Lukacs GL, Frizzell RA.Small heat shock proteins target mutant cystic fibrosis transmembrane conductance regulator for degradation via a small ubiquitin-like modifier-dependent pathway. Mol Biol Cell. 2013;24(2):74-84.

## Towards New Potential Anti-Inflammatory Therapies

### O16 Enabling pulmonary delivery of siRNA in cystic fibrosis lung inflammation: therapeutic potential of hybrid lipid/polymer nanoparticles

#### Ungaro Francesca^1^ and Merkel Olivia M^2^

##### ^1^Università “Federico II”, Napoli, Italy; ^2^Ludwig-Maximilians Universität, München, Germany

###### **Correspondence:** Ungaro Francesca (francesca.ungaro@unina.it)

**Background and rational**

The down-regulation of genes directly involved in the pathogenesis of severe lung diseases through pulmonary delivery of short RNA fragments, also known as siRNA, has gained recently remarkable research interest, especially in cystic fibrosis (CF). Nevertheless, the unsuccessful history of inhaled siRNA points out the urgent need of an appropriate formulation strategy to move them from the laboratory to the bedside.

**Hypothesis and objectives**

The generation of breakthrough technologies and their translation into new pharmaceutical products is crucial for CF treatment. In this context, the general aim of the present project is the integrated development of inhalable hybrid nanoparticles (hNPs) for siRNA delivery made up of a combination of lipids and polymers. To allow a proof-of-concept of the soundness of this approach, the in vitro/in vivo therapeutic potential of hNPs delivering a siRNA against one of the most critical signals in evoking the inflammatory response in CF, the nuclear factor-κB (NF-κB), has been assessed.

**Essential methods**

hNPs delivering a siRNA pool against NF-κB have been prepared from biodegradable polymers and endogenous phospholipids. Toxicity, uptake and efficacy of siRNA-loaded hNPs have been evaluated in different human airway cell culture models, providing a tool to optimise hNP properties for in vivo pulmonary delivery. In vivo studies have been performed in rats challenged intratracheally with LPS from E. Coli to induce pulmonary inflammation.

**Results**

Lipid/polymer hNPs for sustained release at lungs of a siRNA pool against NF-κB have been successfully developed. The most adequate formulation conditions to produce non-PEGylated and PEGylated siRNA-loaded hNPs with optimal aerosolization and mucus-penetrating properties have been identified. Preliminary in vitro data suggest that siRNA-loaded hNPs are not cytotoxic and may penetrate lung extracellular barriers, allowing siRNA uptake inside human bronchial epithelial cells. Furthermore, a rat model of lung inflammation has been set up and validated to start with in vivo efficacy studies.

**Conclusions**

The correct operating conditions to produce nanoparticles for prolonged release of siRNA in CF have been identified, providing a siRNA delivery system already engineered for in vivo inhalation and transfection.

**Acknowledgment**

This study was supported by Italian Cystic Fibrosis Research Foundation (FFC), Research project Number FFC#23/2017

### O17 Pro-resolutive actions of Resolvin D1 in cystic fibrosis

#### Antonio Recchiuti ^1,3^, Veronica Cecilia Mari ^1,3^, Elisa Isopi ^1,3^, Domenico Mattoscio ^1,3^, Alessia Lamolinara ^2,3^, Marco D’Aurora ^4,3^, Valentina Gatta ^4,3^, Manuela Iezzi ^2,3^, Alessandra Bragonzi ^5^, Paolo Moretti ^6^, Marc Dubordeau ^7^, Marilina Codagnone ^1,3^, Eleonora Cianci ^1,3^, Mario Romano ^1,3^

##### ^1^Department of Medical, Oral, and Biotechnology Science University of Chieti-Pescara, Chieti, Italy; ^2^Department of Medicine and Aging Science University of Chieti-Pescara, Chieti, Italy; ^3^Center on Aging Sciences and Translational Medicine (CeSI-MeT) “G. d’Annunzio” University of Chieti-Pescara, Chieti, Italy; ^4^Department of Psychological, Humanistic and Territorial Sciences University of Chieti-Pescara, Chieti, Italy; ^5^Infection and Cystic Fibrosis Unit, Division of Immunology, transplantation, and Infectious Diseases, IRCCS San Raffaele Scientific Institute, Milan, Italy; ^6^Centro Regionale Fibrosi Cistica, Ospedale San Salvatore, Atri (TE), Italy; ^7^Ambiotis SAS, Toulouse, France

###### **Correspondence:** Antonio Recchiuti (a.recchiuti@unich.it)

**Backgroung and rationale**

Non resolving lung inflammation and infections are a main cause of morbidity and mortality in cystic fibrosis (CF)[1-3]. Resolvin (Rv) D1 is an endogenous lipid autacoid that promotes resolution in experimental and human inflammatory diseases [4,5].

Results from a previous FFC-funded research (FFC#21/2014) demonstrate that RvD1: a) reduces inflammation and P. aeruginosa (PA) burden in vivo; b) stimulates microbial clearance by murine and human macrophages; c) protects lungs following persistent P. aeruginosa infection.

Therefore, determining roles and functions of RvD1 in chronic lung inflammation, infection, and damage is of wide interest for CF.

**Hypothesis and Objectives**

To test the hypothesis that RvD1 is effective in promoting resolution of inflammation, microbial clearance, and airway tissue regeneration in CF, two specific aims were addressed:

1. Investigating if RvD1 limits airway chronic inflammation, P. aeruginosa (PA) infection, and damage stimulating tissue repair in preclinical models of CF.
2. Establishing if RvD1 regulates select genes that limit inflammation and promote tissue repair by human CF cells.

**Essential methods**

RvD1 actions were tested in Cftr^-/-^ mice chronically infected with a clinical strain of PA by assessing bacterial burden, inflammation, and tissue damage. Direct actions of RvD1 on primary CF airway epithelial cells (CFAEC) and macrophages (MΦ) were established to pinpoint molecular mechanisms activated by RvD1 to reduce inflammation and enhance resolution.

**Preliminary results (personal)**

RvD1 significantly dampened bacterial burden and LPS amounts in lungs. RvD1 also markedly dampened total leukocytes and PMN infiltration and ameliorated histological scores of lung pathology. In addition, RvD1 increased the percentage of phagocytosis of PA by CF mouse and human CF macrophages along with a significant reduction in interleukin-8, -6, and -17. In CFAEC and MΦ from CF volunteers RvD1 regulated genes involved in inflammation and anti-microbial responses.

**Spin-off for research & clinical purposes**

These results indicate that RvD1 enhances resolution of PA infection and inflammation in CF, thus fostering further studies in preclinical models and clinical trials, as well as spin-off for research applications in a relatively short time (~3-5 years) for exploring RvD1 as a novel therapeutic for CF.

**Acknowledgment** This study was supported by Italian Cystic Fibrosis Research Foundation (FFC), Research project Number FFC#19/2016

**References**

1. Nichols DP, Chmiel JF. Inflammation and its genesis in cystic fibrosis. Pediatr Pulmonol. United States. 2015;50 Suppl 4:S39-56. PMID: 26335954
2. Karp CL, Flick LM, Park KW, Softic S, Greer TM, Keledjian R, Yang R, Uddin J, Guggino WB, Atabani SF, Belkaid Y, Xu Y, Whitsett JA, Accurso FJ, Wills-Karp M, Petasis NA. Defective lipoxin-mediated anti-inflammatory activity in the cystic fibrosis airway. Nat Immunol. 2004;5(4):388–92.
3. Mattoscio D, Evangelista V, De Cristofaro R, Recchiuti A, Pandolfi A, Di Silvestre S, Manarini S, Martelli N, Rocca B, Petrucci G, Angelini DF, Battistini L, Robuffo I, Pensabene T, Pieroni L, Furnari ML, Pardo F, Quattrucci S, Lancellotti S, Davì G, Romano M, Davi G, Romano M. Cystic fibrosis transmembrane conductance regulator (CFTR) expression in human platelets: impact on mediators and mechanisms of the inflammatory response. FASEB J. 2010/06/10. 2010;24(10):3970–3980.
4. Codagnone M, Cianci E, Lamolinara A, Mari VC, Nespoli A, Isopi E, Mattoscio D, Arita M, Bragonzi A, Iezzi M, Romano M, Recchiuti A. Resolvin D1 enhances the resolution of lung inflammation caused by long-term Pseudomonas aeruginosa infection. Mucosal Immunol. United States; 2018;11(1):35–49.
5. Serhan CN, Levy BD. Resolvins in inflammation: emergence of the pro-resolving superfamily of mediators. J Clin Invest. United States; 2018; PMID: 29757195

## Anti-Inflammatories with CFTR Recovery Action

### O18 New generation trimethylangelicin (TMA) analogues for selective modulation of defective CFTR or inflammation

#### Adriana Chilin ^1^, Roberto Gambari ^2^, Ilaria Lampronti^2^, Giulio Cabrini^3^, Maria Cristina Dechecchi^4^

##### ^1^Dipartimento di Scienze del Farmaco, Università di Padova, Padova, Italy; ^2^Dipartimento di Scienze della vita e biotecnologie, Università di Ferrara, Ferrara, Italy; ^3^Dipartimento di Neuroscienze, Biomedicina e Movimento, Università di Verona, Verona, Italy; ^4^Laboratory of Molecular Pathology, Department of Pathology and Diagnostics, University Hospital of Verona, Verona, Italy

###### **Correspondence:** Adriana Chilin (adriana.chilin@unipd.it)

**Background and rationale**

TMA (4,6,4’-trimethylangelicin) is a potentiator and corrector of mutated CFTR protein, with anti-inflammatory activity useful for treating CF lung disease [1,2]. Concerns raised on the potential phototoxicity and mutagenicity of the parent TMA molecule led to design and study new derivatives with one or more of the three biological effects of TMA but excluding the potential safety risks.

**Hypothesis and Objectives**

The major objective of the project was to synthesize and test new generation TMA analogues as F508del CFTR correctors and/or F508del CFTR potentiators and/or inflammatory down- modulators, lacking phototoxicity and mutagenicity. The collected data could have allowed to derive structure-activity relationships aimed at a comprehension of the structural features on the TMA scaffold, required to obtain selective anti-inflammatory or CFTR modulatory properties or both activities.

These new findings could improve CF therapy, providing the option of associating an anti-inflammatory effect besides the key activity of rescuing and potentiating CFTR.

**Essential methods**

The project was realized through the following steps: design and synthesis of new TMA analogues; evaluation of the phototoxicity and mutagenicity; test of the anti-inflammatory activity, test of the effects on CFTR function; derivation of structure-activity relationships.

**Results**

A small library of about 50 new TMA analogues were synthesized with structural modification in the 4 and 6 positions of the furocoumarin nucleus. Among them, some compounds were identified exhibiting CFTR modulation and/or NF-kB inhibition, without the side effects of parent TMA [1]. They maintained the potentiation activity of CFTR and significantly rescued CFTR-dependent chloride efflux in several cell models [2]. These analogues mediated CFTR correction by modifying MSD1 and indirectly stabilizing the interface between NBD1 and CL4.

**Conclusions**

New generation TMA analogues were obtained to overcome the side effects of the parent TMA, maintaining CFTR modulation and/or anti-inflammatory properties. The main structural determinants that drive the biological activities of the parent TMA were determined, thus allowing to find novel candidates with useful features and negligible side effects for pre-clinical studies.

**Acknowledgment**

This study was supported by Italian Cystic Fibrosis Research Foundation (FFC), Research project Number FFC#1/2016

**References**

1. Marzaro G, Lampronti I, D'Aversa E, Sacchetti G, Miolo G, Vaccarin C, Cabrini G, Dechecchi MC, Gambari R, Chilin A. Design, synthesis and biological evaluation of novel trimethylangelicin analogues targeting nuclear factor kB (NF-kB). Eur J Med Chem. 2018; 151:285-293.
2. Laselva O, Marzaro G, Vaccarin C, Lampronti I, Tamanini A, Lippi G, Gambari R, Cabrini G, Bear CE, Chilin A, Dechecchi MC. Molecular Mechanism of Action of Trimethylangelicin Derivatives as CFTR Modulators. Front Pharmacol. 2018; 9:719.

### O19 Anakinra in cystic fibrosis: From targeting pathogenic inflammation to correcting CFTR defects

#### Marilena Pariano^1^, Matteo Puccetti^1^, Vasilis Oikonomou^1^, Monica Borghi^1^, Valeria R. Villella^2^, Claudia Stincardini^1^, Luigi Sforna^1^, Giorgia Renga^1^ and Luigina Romani^1^

##### ^1^Dipartimento di Medicina Sperimentale, Università degli Studi di Perugia, Perugia, Italy; ^2^European Institute for Research in Cystic Fibrosis, Division of Genetics and Cell Biology, San Raffaele Scientific Institute, 20132 Milan, Italy

###### **Correspondence:** Luigina Romani (luigina.romani@unipg.it)

**Background and rationale**

The Phe508del mutation in the first nucleotide-binding domain, which is the most common mutation among individuals with CF, results in the production of a misfolded protein with residual activity that is degraded by the ubiquitin–proteasome system during biogenesis. Thus, regulator of cellular protestasis may alter trafficking of Phe508del-CFTR and favour its plasma membrane targeting and stability.

**Hypothesis and objectives**

In our last CF project, we have defined mechanisms by which dysregulated inflammasome activity may contribute to the vicious circle perpetuating pathogenic inflammation. Anakinra, a recombinant, non-glycosylated version of human IL-1RA, limited the pathological consequences of microbial colonization in CF trough the autophagic/proteasomal degradation system. As this pathway is defective in CF and is associated with failure to target misfolded CFTR for degradation, we have proposed the evaluation of anakinra as a regulator of CFTR protein via proteostasis.

**Essential methods**

The project included murine and human studies.

1. In *vitro*, to assess the effect of anakinra on expression, cellular localization and functional activity of Phe508del-CFTR and the molecular mechanisms behind anakinra’s rescuing activity in Phe508del-CFTR-transfected CFBE41o-cells and HBE cells from patients homozygosus for the Phe508del-CFTR mutation and controls.
2. In *vivo*, in Cftr-Phe508del mice to define the pharmacology of anakinra.

**Results**

The results of our project aimed at elucidating whether anakinra would be capable of promoting CFTR rescuing have indicated that anakinra is able to exert CFTR rescuing activity in Phe508del-CFTR-transfected CFBE41o- cells and HBE cells from CF patients, through both the conventional and unconventional secretion pathways.

**Conclusions**

Studies are underway to define the possible molecular mechanisms underlying the ability of anakinra to promote both pathways. Ultimately, the ongoing studies on the comparative activity of anakinra alone or combined with correctors and/or potentiators on chloride channel activity are definitely required to validate the repurposing of anakinra as therapeutic agent in the real-life CF.

**Acknowledgment**

This study was supported by Italian Cystic Fibrosis Research Foundation (FFC), Research project Number FFC#9/2016

## Antimicrobial Peptides

### O20 Development of inhalable particles for optimal delivery of a potent antimicrobial molecule in *P. aeruginosa* infected lungs

#### Alessandro Pini^1^, Ivana d’Angelo ^2^

##### ^1^Dipartimento di Biotecnologia Medica, Università di Siena; ^2^Dipartimento di Scienze e Tecnologie Ambientali, Biologiche e Farmaceutiche, Di.S.T.A.Bi.F., Seconda Università di Napoli, Napoli, Italy

###### **Correspondence:** Alessandro Pini (pinia@unisi.it)

**Background and rational**

The antimicrobial peptide M33 is a molecule currently in preclinical development for the set up of a new antibiotic for lung infections in Cystic Fibrosis (CF) patients. In parallel to the preclinical development of the molecule as a free drug, we are developing and characterizing optimized drug delivery systems to enhance its effectiveness. Here we designed and developed polymeric nanoparticles (NPs) for the peptide delivery to the lung, in order to achieve an optimized activity of M33 to the pulmonary infection sites. This work was done in collaboration between two research groups from Siena and Caserta.

**Hypothesis and objectives**

The Siena group was dedicated to the molecule production test in vitro and possible animal experiments. The Caserta Unit was dedicated to the selection and characterization of the drug delivery formulations encapsulating M33.

**Essential Methods**

The M33 peptide was produced according to the manufacturing procedures developed in the past, and encapsulated in different polymeric NP formulations, in order to achieve an optimized peptide formulation able to improve the M33 activity. The developed M33-loaded NP formulations were tested in vitro for their efficacy and toxicity.

**Results**

During the project several lots of PLGA-based nanoparticles (NPs) containing M33 were produced. The obtained delivery systems showed optimized size, zeta potential, in vitro aerosolizzation properties and peptide encapsulation and in vitro release. The obtained formulations were tested for antimicrobial activity by MIC assays against P. aeruginosa, and for toxicity against eukaryotic cells. No significant toxicity was revealed.

In parallel, an animal model of lung infection with P. aeruginosa was set up using a Penn Century device for nebulization and an aerosol machine specifically constructed for animal exposure to encapsulated M33.

**Conclusions**

In order to improve M33 delivery we set up a novel NP preparations based on PLGA and containing the peptide M33. The most important results regarded the strong decrease of toxicity with respect of the non-encapsulated peptide. Experiments in vitro were set-up for the measurement of M33 efficacy using culture medium where bacteria grew slowly and the peptide released from NPs could be active against bacteria. An animal model was also set-up for future in vivo experiments.

**Acknowledgment**

This study was supported by Italian Cystic Fibrosis Research Foundation (FFC), Research project Number FFC#17/2016

### O21 Frog skin-derived peptides for treatment of *Pseudomonas aeruginosa* lung infection and bronchial epithelial repair: advanced in vitro and in vivo characterization and development of polymeric nanoparticles for lung delivery

#### Maria Luisa Mangoni^1^, Bruno Casciaro^1^, Ivana d’Angelo^2^, Xiaoping Zhang^3^, Floriana Cappiello^1^, Mariarosa Loffredo^1^, Peter Y. Di^3^, Francesca Ungaro^4^

##### ^1^Laboratory affiliated to Pasteur Italia-Fondazione Cenci Bolognetti, Department of Biochemical Sciences, Sapienza University of Rome, Rome, via degli Apuli, 9,00185, Italy; ^2^Di.S.T.A.Bi.F., University of Campania "Luigi Vanvitelli", Via Vivaldi 43, 81100 Caserta, Italy; ^3^Department of , Environmental and Occupational Health, University of Pittsburgh, Pittsburgh, PA, 15260, USA; ^4^Department of Pharmacy, University of Naples Federico II, Via D Montesano 49, 80131 Naples, Italy

###### **Correspondence:** Maria Luisa Mangoni (marialuisa.mangoni@uniroma1.it)

**Background and rationale**

*Pseudomonas aeruginosa* is the most predominant pulmonary pathogen in cystic fibrosis (CF)[1]. It is quite difficult to eradicate, mainly due to its resistance to most available antibiotics and ability to form biofilms. We identified a peptide from frog skin (21 amino acids), Esc(1-21), which rapidly kills *P. aeruginosa* with a membrane-perturbing activity that prevents bacteria from developing resistance[2]. Recently, we designed a diastereomer of Esc(1-21), more stable and less cytotoxic than the wild-type peptide; more efficient in stimulating migration of bronchial cells and presumably in promoting recovery of the bronchial epithelium integrity[3,4]. This is a relevant feature considering the defective airway epithelial wound repair in CF sufferers. Yet, the diastereomer was more efficient in reducing lung bacterial burden in mouse models of *P. aeruginosa* lung infection. However, conceiving AMPs for local delivery to the lungs, adequate airway delivery strategies are needed to promote their transport to the infection site.

**Hypothesis and objectives**

Main objectives of the Project were (i) an in-depth study of the re-epithelialization activity of these peptides and (ii) the development of new effective and economically feasible polymeric nanoparticles (NPs) loaded with each peptide to assist its diffusion through the bronchial mucus and to allow local treatment of *P. aeruginosa* lung infection.

**Essential methods**

A multidisciplinary approach combining biochemical, microbiological techniques and preclinical testing in mouse models to explore the efficacy and safety profile of the selected peptides and peptide-loaded NPs.

**Results**

Preclinical data in a murine model of *P. aeruginosa* lung infection have provided the first evidence of the success of poly lactic-co-glycolic NPs as valuable nanocarriers to assist the delivery of antimicrobial peptides in the conductive airways as well as to boost up their antimicrobial effect. Furthermore, we have observed that the re-epithelialization of the bronchial epithelium by these peptides involves transactivation of epidermal growth factor receptor.

**Conclusions**

On the basis of our findings, it can be concluded that the diastereomer represents the best candidate for the development of inhalable nanoformulations for controlled delivery of the peptide at CF lungs prolonging its therapeutic efficacy with minimal side-effects. These NPs could be further developed into dry powders for inhalation allowing a much easier and faster administration as compared to nebulized liquid formulations or intravenous injection.

**Acknowledgment**

This study was supported by Italian Cystic Fibrosis Research Foundation (FFC), Research project Number FFC#15/2017

**References**

1. Bjarnsholt T, Jensen PØ, Fiandaca MJ, Pedersen J, Hansen CR, Andersen CB, Pressler T, Givskov M, Høiby N. Pseudomonas aeruginosa biofilms in the respiratory tract of cystic fibrosis patients. Pediatr Pulmonol. 2009;44(6):547-58.
2. Luca V, Stringaro A, Colone M, Pini A, Mangoni ML. Esculentin(1-21), an amphibian skin membrane-active peptide with potent activity on both planktonic and biofilm cells of the bacterial pathogen Pseudomonas aeruginosa. Cell Mol Life Sci. 2013;70(15):2773-86.
3. Di Grazia A, Cappiello F, Cohen H, Casciaro B, Luca V, Pini A, Di YP, Shai Y, Mangoni ML. D-Amino acids incorporation in the frog skin-derived peptide esculentin-1a(1-21)NH2 is beneficial for its multiple functions. Amino Acids. 2015;47(12):2505-19.
4. Cappiello F, Di Grazia A, Segev-Zarko LA, Scali S, Ferrera L, Galietta L, Pini A, Shai Y, Di YP, Mangoni ML. Esculentin-1a-Derived Peptides Promote Clearance of Pseudomonas aeruginosa Internalized in Bronchial Cells of Cystic Fibrosis Patients and Lung Cell Migration: Biochemical Properties and a Plausible Mode of Action. Antimicrob Agents Chemother. 2016;60(12):7252-7262.

### O22 Pre-clinical effectiveness of three human cryptic antibiofilm peptides (GVF27, HVA36 and IMY47): efficacy against lung pathogens and studies in animals

#### Andrea Bosso^1^, Rosa Gaglione^2^, Siepi Maria Luisa^1^, Valeria Cafaro^1^, Eugenio Notomista^1^, Angela Arciello^2^, Eliodoro Pizzo^1^

##### ^1^Dipartimento di Biologia, Università di Napoli Federico II, Napoli, Italy; ^2^Dipartimento di Scienze Chimiche, Università di Napoli Federico II, Napoli, Italy

###### **Correspondence:** Eliodoro Pizzo (elipizzo@unina.it)

**Background and Rationale**

Microbial biofilms are the underlying cause of persistent infections in Cystic Fibrosis lungs. The formation and maintenance of biofilms depends critically on the presence of bacteria-to-bacteria interconnecting extracellular substances that serve as a biofilm matrix. Biofilms display a high resistance to killing by most antimicrobial compounds and this high level of resistance depends on multiple factors associated with adaptive changes in gene expression accompanying the biofilm growth state and the inherent properties of biofilm structures that act as physical barrier to antibiotic penetration. For this reason new drugs, alternative to conventional antibiotics, are needed to combat specifically biofilm forming pathogens. At this regard, Host Defence Peptides (HDPs), are a valuable attractive both for their selective toxicity and multi-immunomodulatory properties. During FFC project #20/2014 we have identified [1] and studied several new human cryptic HDPs never analysed so far. Some of these molecules have interesting features [2-4] and for this reason we have deepened their study in the perspective as new potential CF therapeutics.

**Hypothesis and Objectives**

The aim of our pilot project FFC project #16/2017, was to characterize three very promising peptides (GVF27, HVA36 and IMY47) for their antimicrobial and anti-biofilm properties, alone or by combination therapy approaches, as well as analyse their possible biocompatibility on cultured epithelial cells and *in vivo* on murine models.

**Essential Methods**

The three peptides have been extensively characterized for their activities, alone or in combination with antibiotics, on a broad spectrum of CF clinical isolates. We have also evaluated their anti-biofilm properties (by static and co-culture experiments), their affinity for endotoxins (e.g. LPS) by CD analysis, their immunomodulatory properties (immune-enzymatic assays) on LPS induced murine macrophages and bronchial epithelial cells and their toxicity *in vivo* on mice by CFaCore facility.

**Results**

All three cryptic peptides show significant antimicrobial activities (with MIC ranging from 2 to 10 μM) on CF clinical isolates, good affinity for LPS (HVA36 and GVF27) and LTA (GVF27) endotoxins, and significant antibiofilm properties (GVF27 and HVA36). Nevertheless IMY 47 is the only non-toxic when administered in vivo (by aerosol treatments and also by cutaneous injections) and moreover it presents in its sequence a cryptic potent anti-biofilm peptide (IMY25) that we have successfully produced and in vitro characterized. Starting from these observations, our future efforts will be dedicated to explore their therapeutic potentialities in cell-based and pre-clinical models of CF.

**Conclusions**

The development of novel therapies represents a priority to relieve life conditions of CF patients. Our studies point to potential use of novel anti-biofilm drugs of human origin, alone or combined with antibiotics, could crucially improve the treatment to eradicate bacterial lung invasion.

**Acknowledgment**

This study was supported by Italian Cystic Fibrosis Research Foundation (FFC), Research project Number FFC#16/2017

**References**

1. Pane K, Durante L, Crescenzi O, Cafaro V, Pizzo E, Varcamonti M, Zanfardino A, Izzo V, Di Donato A, Notomista E. Antimicrobial potency of cationic antimicrobial peptides can be predicted from their amino acid composition: Application to the detection of "cryptic" antimicrobial peptides. J Theor Biol. 2017;419:254-265.
2. Pane K, Cafaro V, Avitabile A, Torres MT, Vollaro A, De Gregorio E, Catania MR, Di Maro A, Bosso A, Gallo G, Zanfardino A, Varcamonti M, Pizzo E, Di Donato A, Lu TK, de la Fuente-Nunez C, Notomista E. Identification of Novel Cryptic Multifunctional Antimicrobial Peptides from the Human Stomach Enabled by a Computational-Experimental Platform. ACS Synth Biol. 2018;7(9):2105-2115.
3. Gaglione R, Dell'Olmo E, Bosso A, Chino M, Pane K, Ascione F, Itri F, Caserta S, Amoresano A, Lombardi A, Haagsman HP, Piccoli R, Pizzo E, Veldhuizen EJA, Notomista E, Arciello A Novel human bioactive peptides identified in Apolipoprotein B: Evaluation of their therapeutic potential. Biochem Pharmacol. 2017;130:34-50.
4. Bosso A, Pirone L, Gaglione R, Pane K, Del Gatto A, Zaccaro L, Di Gaetano S, Diana D, Fattorusso R, Pedone E, Cafaro V, Haagsman HP, van Dijk A, Scheenstra MR, Zanfardino A, Crescenzi O, Arciello A, Varcamonti M, Veldhuizen EJA, Di Donato A, Notomista E, Pizzo E. A new cryptic host defense peptide identified in human 11-hydroxysteroid dehydrogenase-1 β-like: from in silico identification to experimental evidence. Biochim Biophys Acta Gen Subj. 2017;1861(9):2342-2353.

## Non-Tubercolous mycobacteria and Aspergillus in Cystic Fibrosis

### O23 Identification of new efflux pumps inhibitors able to contrast nontuberculous mycobacterial infections in cystic fibrosis patients

#### Rolando Cannalire,^1^ Tommaso Felicetti,^1^ Andrea Astolfi,^1^ Maria Letizia Barreca,^1^ Giuseppe Manfroni,^1^ Aurélie Marie-Madeleine Schoubben,^1^ Violetta Cecchetti,^1^ Miguel Viveiros,^2^ Stefano Sabatini^1^

##### ^1^Department of Pharmaceutical Sciences, University of Perugia, via del Liceo 1, 06123 Perugia, Italy; ^2^Unidade de Microbiologia Medica, Global Health and Tropical Medicine, GHTM, Instituto de Higiene e Medicina Tropical, IHMT, Universidade NOVA de Lisboa, UNL, Rua da Junqueira 100, 1349-008 Lisboa, Portugal

###### **Correspondence:** Stefano Sabatini (stefano.sabatini@unipg.it)

**Background and rationale**

Non-tuberculous mycobacteria (NTM) are very difficult to eradicate pathogens and in cystic fibrosis (CF) patients are often associated to serious chronic infections (about 10%). M. avium causes almost half of the infections and rapidly develops resistance due to the overexpression of efflux pumps (EPs) able to extrude from the cell antimycobacterial agents such as macrolides. Therefore, inhibiting EPs is a promising strategy to contrast these infections, restoring the sensitivity of mycobacteria to ineffective drugs and preventing the microorganism from developing specific resistance mechanisms.

**Hypothesis and objectives**

The main aim of the project was the development of M. avium EP inhibitors (EPIs) able to synergize the activity of antibacterial agents that are now obsolete and thus open the way to a new anti-infective strategy that can improve the lifestyle of CF patients.

**Essential methods**

In this project, by exploiting a multidisciplinary approach including drug-design, chemical synthesis and biological evaluations, we performed the chemical optimization of compounds with a 3-phenylquinolone scaffold previously identified by us as M. avium EPIs. The compounds designed and synthesized have been preliminarily tested against M. smegmatis to evaluate EPI activity and on human cells (macrophages) to determine toxicity. The best compounds in terms of toxicity/activity ratio have been advanced against M. avium.

**Results**

Our studies have led to the identification of some new molecules with a high inhibitory potency of EPs and one of these in particular is able to enhance the activity of antibiotics such as clarithromycin, which is a drug of choice for the NTM infections treatment, and ciprofloxacin against M. avium, at lower concentrations than those toxic to macrophages.

**Conclusions**

Considering the field of NTMs EPIs, this compound has the best toxicity/activity profile ever shown and can represent a new starting point for further optimization aimed at identifying preclinical candidates.

**Acknowledgment**

This study was supported by Italian Cystic Fibrosis Research Foundation (FFC), Research project Number FFC #17/2017

### O24 Establishment of animal model to investigate pathogenesis of infection by *Mycobacterium abscessus* complex members in cystic fibrosis patients

#### Camilla Riva^1^, Floriana Gona^1^, Marco Rossi^1^, Cristina Cigana^2^, Alessandra Bragonzi ^2^, Daniela M Cirillo^1^, Enrico Tortoli^1^

##### ^1^Emerging Bacterial Pathogens Unit, IRCCS San Raffaele Scientific Institute, Milano, Italy; ^2^Infections and Cystic Fibrosis Unit, IRCCS San Raffaele Scientific Institute, Milano, Italy

###### **Correspondence:** Enrico Tortoli (tortoli.enrico@hsr.it)

**Background and rationale**

*M. abscessus* (MA) is one of the most frequently isolated non tuberculous mycobacteria (NTM) in patients with cystic fibrosis (CF)[1]. Despite the reports on increasing prevalence of MA, including multidrug resistant strains, its pathogenic role is still controversial, due to the limitations of the available cellular and animal models used to study MA infection[2].

**Hypothesis and objectives**

Our hypothesis is that strains of MA isolated from patients with deteriorated lung functionality may differ in pathogenicity from the ones isolated from asymptomatic patients. For this reason we investigated the pathogenicity of the MA subspecies (subs) to identify the patients who could benefit from antimicrobial treatment.

**Methods**

MA subs reference strains (MA *abscessus*, MA *bolletii* and MA *massiliense*) and isolates from CF patients were used to establish chronic infection, using the agar beads method[3], in WT and CF mice up to six months. Pulmonary mice lesions were monitored by magnetic resonance imaging (MRI) and at different time points mice lungs were processed for microbiological analysis, inflammatory response and histological evaluation.

**Results**

Using different reference and clinical strains of MA subs we were able to establish a long-term (up to 3 months) chronic lung infection in WT and CF mice with a stable bacterial load ( ~1x10^5^ CFU). After 6 months of MA infection the lung was still characterized by a granulomatous response with aggregation of lymphocytes and macrophages despite, at this time point, some animals had cleared the lung infection. The persistence of lung lesions was also confirmed by the quantification of the infectious foci made possible with MRI. The inflammatory response in bronchoalveolar lavage fluid was not statistically different from the one of control mice while in total lung the level of cytokines/chemokines (TNF-α, IFN-γ, GM-CSF, IL-1β and KC) was sustained during the all course of MA infection.

**Conclusions**

We could therefore establish, for the first time, a model of chronic MA lung infection with minimal systemic involvement in in immune-competent mice. The availability of this murine model and the longitudinal MRI monitoring of mice will hopefully allow to identify the MA isolates responsible for severe disease and to investigate in vivo the impact of novel therapeutic protocols.

**Acknowledgment**

This study was supported by Italian Cystic Fibrosis Research Foundation (FFC), Research project Number FFC#20/2017

**References**

1. de Vrankrijker AM1, Wolfs TF, van der Ent CK. Challenging and emerging pathogens in cystic fibrosis. Paediatr Respir Rev. 2010;11(4):246-54
2. Ordway D, Henao-Tamayo M, Smith E, Shanley C, Harton M, Troudt J, Bai X, Basaraba RJ, Orme IM, Chan ED. Animal model of Mycobacterium abscessus lung infection. Leukoc Biol. 2008; 83(6):1502-11
3. Cigana C, Lorè NI, Riva C, De Fino I, Spagnuolo L, Sipione B, Rossi G, Nonis A, Cabrini G, Bragonzi A.Tracking the immunopathological response to Pseudomonas aeruginosa during respiratory infections. Sci Rep. 2016;6:21465.

## Corrective Approaches for Non-F508DEL-CFTR Mutations and Gene-cell therapy

### O25 Dissecting the potency of human Mesoangioblasts to differentiate into CFTR-expressing epithelial cells: a step forward to an innovative cell-based therapy for Cystic Fibrosis disease

#### Graziella Messina^1^, Nicoletta Pedemonte^2^

##### ^1^Dipartimento di Bioscienze, Università degli Studi di Milano, Milano, Italy; ^2^Istituto G. Gaslini, U.O.C. Genetica Medica, Genova, Italy

###### **Correspondence:** Graziella Messina (graziella.messina@unimi.it)

**Background**

Since the CFTR gene was cloned in 1989, several strategies for correction of CF lung disease have been explored. Among these, cell-based approaches are under investigation. Endogenous lung stem and progenitor cells have been studied although their contribution in the amelioration of chronic lung diseases is still debated. Stem cell-based approaches to treat CF have not achieved the efficiencies of delivery and engraftment needed for therapy. The reasons rely on the low cell engraftment in the lungs after systemic administration and on the small percentage of differentiated cells in airway epithelia and in the even less percentage of CFTR expression.

**Hypothesis and objectives**

During our recent works on a class of mouse progenitor cell derived from vessel, named mesoangioblasts (mMABs), we observed that mMABs, when systemically transplanted in healthy and CF mice distributed throughout lung, trachea and intestinal epithelium over-time. The aim of this project was to evaluate for the first time the potency of human mesoangioblast to correct the CFTR defect. The whole study can be considered a crucial step to definitively develop a cell-based therapy for patients affected by CF.

**Essential methods**

Different populations of human MABs (hMABs) have been tested for the expression of CFTR. hMABS have been co-cultured with human bronchiolar epithelial cells from CF patients bearing severe mutations of CFTR to evaluate their ability to differentiate into epithelium and to express a functional CFTR (by Ussing chamber), thus mimicking the in vivo environment.

**Results**

We observed that hMABs already in vitro express, although at low levels, both the immature and mature forms of CFTR. Notably, this expression corresponds, in ex vivo Ussing chamber by co-cultures of hMABs with CF bronchiolar epithelial cells, to a functional CFTR channel.

**Conclusions**

This project represents a major advance over any other cell-based therapeutic strategy for CF. This first study on human MABs demonstrates their ability to express functional CFTR, rescuing chloride ions transport in CF epithelia and possibly differentiating into epithelial like cells. The results obtained from this proposal will definitively make these cells eligible for a clinical translation in human CF patients as a cell based therapy.

**Acknowledgment**

This study was supported by Italian Cystic Fibrosis Research Foundation (FFC), Research project Number FFC#5/2017

## In Vivo, Ex Vivo and in Vitro Predictive Tests and Models to Evaluate the CFTR function

### O26 Implementation of a new imaged-controlled sweat test for in vivo quantification of CFTR function: value for diagnosis and efficacy of new therapies

#### Teresinha Leal^1^, Stefano Ceri ^2^, Nguyen-Khoa Thao ^3^

##### ^1^Louvain Centre for Toxicology and Applied Pharmacology, UC Louvain, Louvain, Belgium; ^2^Dipartimento di Elettronica, Informazione e Bioingegneria, Università di Milano, Milano, Italy; ^3^Necker-Enfants Malades Hospital, AP-HP Laboratory of General Biochemistry, Paris, France

###### **Correspondence:** Teresinha Leal (teresinha.leal@uclouvain.be)

**Background and rationale**

A new generation β-adrenergic-dependent sweat secretory test based on imaging of droplets of sweat formed at the surface of the skin (bubble test) was developed in Stanford.

**Hypothesis and objectives**

The main goal of the project was to build a cross-university network (Verona, Brussels and Paris) allowing spreading the bubble test out. The main goal of the Verona CF center was to implement the test in Europe. The main goal of the Brussels center was to develop a non-invasive version of the test, without intradermal injections. That of the Paris center was to make comparisons with the Toronto’s evaporimetry version of the β-sweat secretory test for which the center has acquired a vast experience.

**Essential methods**

Adapting and updating setup and materials of the bubble test method previously described and following a well-established protocol for the evaporimetry method.

**Results**

The Verona center has recently published data confirming the approximately linear readout of CFTR function obtained in tests performed in three groups (CF subjects, CF carriers and non-CF controls, n = 22 in each group). Results showed that all groups were clearly discriminated, with sensibility and specificity ranging from 82% to 100%. The Verona group recently reported data on a new Optical Sweat Rate Beta Adrenergic (OSRBA) test for measuring sweat rates in individual human sweat glands based on a multilinear regression model. It showed that the volume of sweat secretory glands discriminated between CFTR genotypes and allowed quantifying efficacy of pharmacological treatments with CFTR modulators; *i.e.* lumacaftor/ivacaftor (Orkambi) or PTC 124 (Ataluren). The Brussels center had success in the development of a non-invasive version of the test by iontophoresis of the pharmacological agents used, which is particularly challenging for those triggering the β-adrenergic phase of the test. Interim results obtained from 10 patients with CF, 29 healthy subjects and 1 CF carrier confirmed the good discriminative power of the test. Approval by the Ethics committee to conduct the comparative trial was obtained in the Paris center and solutions for injections are managed by the local institutional Pharmacy.

**Conclusions**

We have confirmed during this project that, either under intradermal injections or iontophoresis, the sweat droplet (bubble) test is able to fully discriminate between CF, non-CF and healthy subjects. Further comparative studies between measurements of bubble volumes and of water evaporated during sweat are ongoing.

**Acknowledgment**

This study was supported by Italian Cystic Fibrosis Research Foundation (FFC), Research project Number FFC**#**5/2016

### O27 Human intestinal organoids for detecting CFTR rescue by molecules targeting CFTR mutations in human plasma samples

#### Paola Melotti (paola.melotti@ospedaleuniverona.it)

##### Centro Fibrosi Cistica Azienda Ospedaliera Universitaria Integrata Verona, Verona, Italy

**Background and rationale**

Active drugs on certain mutations of the CFTR gene are now available for CF therapy. Many drugs in the circulation can be inactivated by plasma proteins regulated by various nutritional, infectious, and inflammatory conditions. This exposes to variability of response to the same drug among different patients.

**Hypotheses and objectives**

To investigate the variability of response among CF patients, the levels of active circulating plasma drug of patients undergoing treatment were assessed, which can be detected by the swelling of human intestinal organoids, structures derived from rectal mucosa, available in the laboratory for long periods.

**Essential methods**

Active drugs were analyzed on specific CFTR gene mutations in the plasma of CF patients during therapy, in particular Ivacaftor / Lumacaftor (VX-770 / VX-809, Orkambi). With the known dose of drug added to the human plasma laboratory of non-CF volunteers, the method was set up. The swelling effect on the organoids is compared with the plasma effect of CF patients during treatment.

**Results**

Patients with circulating levels of Ivacaftor / Lumacaftor have been identified as effective in inducing the swelling of the organoids. In these conditions the clinical recovery was not identifiable with spirometry because the pulmonary function was either too compromised or almost normal; chlorine in the sweat expressing CFTR function had changed differently in individual patients during treatment without normalizing.

**Conclusions**

This study found in plasma of CF patients in therapy with Ivacaftor / Lumacaftor the presence of effective drug in correcting the function of CFTR defective in CF organoids homozygous for the F508del mutation.

Personalized medicine becomes possible with this model of organoids, even more considering that the plasma could be tested in organoids derived from rectal biopsies of the same patient (obtained with a non-disturbing, painless procedure). The organoids are thus used completely for the development of new drugs, prediction of clinical response, monitoring of therapies established in individual patients, diagnosis of atypical forms and greater understanding of the consequences of different mutations of the CFTR gene.

**Acknowledgment**

This study was supported by Italian Cystic Fibrosis Research Foundation (FFC), Research project Number FFC#7/2016

